# Augmenting Head and Neck Multidisciplinary Tumor Board Recommendations With Locally Run Large Language Models: Prospective Evaluation of Real-World Implementation

**DOI:** 10.2196/95081

**Published:** 2026-08-20

**Authors:** Christoph Raphael Buhr, Lukas Müller, Daniel Pinto dos Santos, Daniel Thiem, Jean-Jacques Ponciano, Maximilian Krüger, Karoline O'Brien, Justus Kaufmann, Hildegard Nolte, Martin Gartenschläeger, Stefanie Zimmer, Sebastian Altmann, Andrew Blaikie, Christian Ruckes, Christoph Matthias, Sebastian Kuhn, Jonas Eckrich

**Affiliations:** 1 Department of Otorhinolaryngology, University Medical Center of the Johannes Gutenberg-University Mainz, Langenbeckstraße 1, Mainz, Germany, 49 6131 17 7362; 2 University of St Andrews, School of Medicine, St Andrews, United Kingdom; 3 Department of Diagnostic and Interventional Radiology, University Medical Center of the Johannes Gutenberg-University Mainz, Mainz, Germany; 4 Department of Oral and Maxillofacial Surgery-Plastic Surgery, University Medical Center Mainz of the Johannes Gutenberg-University Mainz, Mainz, Germany; 5 Department of Radiotherapy and Oncology, University Medical Center of the Johannes Gutenberg-University Mainz, Mainz, Germany; 6 Department of Hematology & Medical Oncology, University Medical Center Mainz of the Johannes Gutenberg-University Mainz, Mainz, Germany; 7 Institute of Pathology, University Medical Center of the Johannes Gutenberg-University MainzMainz, Germany; 8 Department Neuroradiology, University Medical Center Mainz of the Johannes Gutenberg-University Mainz, Mainz, Germany; 9 Interdisciplinary Center for Clinical Trials (IZKS), University Medical Center of the Johannes Gutenberg-University Mainz, Mainz, Germany; 10 Institute for Digital Medicine Philipps-University Marburg and University Hospital of Giessen and Marburg, Marburg, Marburg, Germany

**Keywords:** large language models, LLM, AI, otorhinolaryngology, ORL, head and neck, digital health, chatbot, language model

## Abstract

**Background:**

Multidisciplinary tumor boards (MDTs) constitute the foundation of modern tumor therapy. Large language models (LLMs) are widely discussed for optimizing their recommendations.

**Objective:**

This is the first prospective feasibility study evaluating the implementation of locally run LLMs on real-world cases within a regular head and neck MDT.

**Methods:**

Seventeen patients participated in the study. The MDT cases were processed by 2 different local LLMs (gemma-3-12b and gpt-oss-20b) to obtain treatment recommendations. The MDT conferred as usual. After the decision was made, the MDT was presented with the LLMs’ recommendations. If deemed to be beneficial, the MDT’s recommendation was adjusted. The MDT members rated the LLMs’ responses inter alia, for medical adequacy on a 6-point Likert scale. In addition, a tabular comparison of the MDT’s and LLMs’ recommendations was carried out.

**Results:**

In one case, 6% (1/17, 95% CI 0%‐29%), the LLM was able to substantially improve the MDT recommendation by underscoring a follow-up examination that had not yet been performed. Concordance regarding the curative or palliative therapy regimen reached 94% (16/17, 95% CI 71%‐100%); for gemma-3-12b and 59% (10/17, 95% CI 33%‐82%) for gpt-oss-20b. Gemma-3-12b stated the same first-line therapy regimen as the MDT as first-line in 35% (6/17, 95% CI 14%‐62%) of cases, and gpt-oss-20b in 41% (7/17, 95% CI 18%‐67%) of cases. In 59% (10/17, 95% CI 33%‐82%) of patients, gemma-3-12b stated the MDT’s first-line therapy regimen, albeit with a different priority, while for gpt-oss-20b, it was 41% (7/17, 95% CI 18%‐67%) of patients. Medical adequacy, as rated by the MDT members, revealed a median of 5 (IQR 2‐5) for gemma-3-12b and 4 (IQR 3‐5) for gpt-oss-20b. MDT members stated potentially hazardous information in 27% (25/93, 95% CI 18%‐37%) of ratings for gemma-3-12b and 17% (14/83, 95% CI 9%‐26%) of ratings for gpt-oss-20b.

**Conclusions:**

Locally run LLMs improved the MDT recommendation in 1 case and were primarily useful for identifying potentially relevant missing information in other cases, underscoring that they cannot replace MDTs. However, their observed benefit suggests that more advanced local models may offer safe, rapid, and cost-effective support for MDT decision-making. The study should be seen as an exploratory setting focusing on practical insights rather than benchmarking its clinical impact. Accordingly, the data demonstrate that the integration of LLMs in today’s MDT workflow is feasible and may benefit the quality of decision-making in specific cases.

## Introduction

### Multidisciplinary Tumor Board

Multidisciplinary tumor board (MDT) recommendations have a central influence on the fate of patients with tumors and form the foundation of therapeutic decision-making in modern tumor therapy. Accordingly, optimizing these treatment decisions is a field of great scientific relevance. In recent years, the use of large language models (LLMs) for MDTs has gained increasing attention.

### LLM Types and Terminology

LLM systems relevant to MDT support can be grouped along three practical dimensions: (1) deployment (cloud-based vs on-premises), (2) model accessibility (closed-weight/vendor models vs open-weight models that can be self-hosted), and (3) model size, where small open-weight models (approximately 7-20B parameters) can often run on standard local hardware, whereas large open-weight models (eg, 70B+parameters) typically require server-grade graphics processing units (GPUs). A further safety-relevant distinction is whether outputs are generated from the model alone (ungrounded), merely instructed to follow guidelines (guideline-instructed), or explicitly grounded in the full text of authoritative guidance (eg, via retrieval-augmented generation that provides the relevant guideline passages at inference time).

### Web-Based LLMs in Head and Neck MDTs

Different studies have evaluated the performance of web-based LLMs in the context of head and neck MDTs [1-4]. Lechien et al [1] assessed ChatGPT-4 on 20 medical records of patients with head and neck cancer regarding additional examinations, management, and therapeutic approaches. The authors report an accurate therapeutic proposition for 65% (13/20) of the cases and a significantly higher number of additional examinations than recommended by practitioners, concluding that LLMs may be an adjunctive theoretical tool in simple oncological board decisions. Schmidl et al [2] shared details of 20 cases from an MDT with ChatGPT 3.5 and ChatGPT 4.0, reporting that the LLMs provided significantly more treatment options than the MDT. However, due to incorrect treatment options in some instances, the authors conclude that the LLMs are currently only suitable as supporting tools. A further study by the same workgroup assessed Claude 3 Opus and ChatGPT 4.0 on primary head and neck cancer cases [3]. Here, the authors describe a similar performance of the models regarding clinical recommendations, explanation, and summarization, but a superior performance of Claude 3 Opus over ChatGPT 4.0 for diagnostic workup and treatment recommendations. Vural Camalan et al [4] compared ChatGPT-o1 and DeepSeek V3 on simulated cases, stating correct treatment recommendations in 62% of cases for ChatGPT-o1 and 80% of cases for DeepSeek V3.

### Addressing Data Protection Aspects by Locally Run LLMs

Data protection regulations, including the Health Insurance Portability and Accountability Act in the United States [5] and the General Data Protection Regulation in Europe [6] play a central role in how sensitive medical data should be processed and protected. These regulatory requirements may pose substantial data protection and compliance challenges for the use of externally hosted LLMs with sensitive clinical data, shifting the focus for real-world clinical applications to locally operated LLMs that comply with data protection requirements by working behind the firewall of health care services. However, there has been little work to date focusing on these locally run LLMs. A study in head and neck cancer compared the performance of web-based and locally operated open-weight LLMs based on simulated cases [7]. Auberville et al [8] demonstrated that alignment methods, in-context learning, and parameter-efficient fine-tuning strongly increased the models’ overall performance on MDT recommendations, reaching congruence of up to 79% for the fine-tuned Mistral 7B.

### Objective of This Study

A review of the literature highlights the potential and limitations of applying LLMs in clinical practice [9-12]. However, promising technology can only make a difference if it is applied in clinical practice. Bridging the gap between simulation and practice, this feasibility study aims to provide the first prospective, real-world evaluation of guideline-instructed, nongrounded, locally operated LLMs as an augmentative tool for the regular MDT in head and neck cancer.

## Methods

### Study Design and Ethics Approval

**Figure 1.**
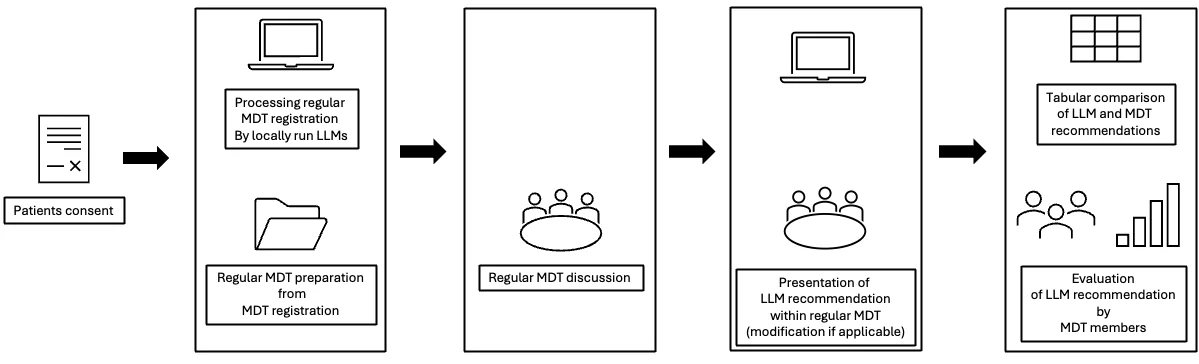
Workflow of the study. LLM: large language models; MDT: multidisciplinary tumor boards.

Ethical approval was obtained from the ethics committee of the state medical association (approval number 2024‐17946_2). The workflow of the study is illustrated in Figure 1.

**Figure 2.**
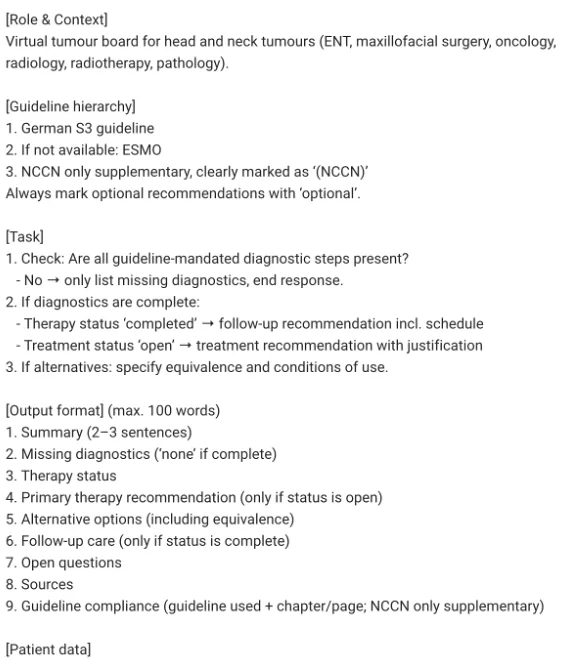
Prompt translated by DeepL (Cologne, Germany) [13]. ENT: Ear Nose Throat; ESMO: European Society for Medical Oncology; NCCN: National Comprehensive Cancer Network.

Informed consent was obtained from patients with a malignant disease who were scheduled to be seen in the MDT for head and neck oncology at our clinic. Information from the regular MDT registration was presented to 2 different locally run LLMs using the prompt shown in Figure 2 ahead of the MDT meeting. We evaluated 2 on-premises small open-weight models (gemma-3-12b and gpt-oss-20b) using a guideline-instructed prompt. The prompt requested guideline-based recommendations following a hierarchy (German S3 guideline as the primary reference, then the European Society for Medical Oncology, with the National Comprehensive Cancer Network only supplementary), but no document-level retrieval of guideline text was implemented; the models therefore relied on their internal knowledge when citing sources.

The MDT meeting was conducted as usual, and MDT members, including specialists in otorhinolaryngology-head and neck surgery, oral and maxillofacial surgery, medical oncology, radiology, radiation oncology, and pathology, did not know in advance which cases were processed by an LLM. After the specific case was discussed within the usual workflow and a decision or recommendation was reached, the MDT was confronted with the recommendations of the 2 LLMs. The recommendations of the 2 LLMs were discussed, and, if applicable, the MDT recommendation was adjusted. This workflow was implemented due to ethical considerations, which were explicitly required by the ethics committee. The requirements stated that the MDT’s decision should not be primarily influenced by the LLM, but merely optimized where possible. The ethics committee permitted corrections of the MDT recommendation only if the LLM raised a point that the MDT had, in fact, overlooked or misjudged. Furthermore, MDT members were asked independently to complete a short questionnaire. The questionnaire was anonymous, and responses could be submitted either digitally (LimeSurvey GmbH) or on paper. For each patient, a separate survey was conducted in which participants rated the recommendations of both LLMs separately. Participants could either complete the questionnaire straight away or after the MDT. The questions did not include any forced-choice options, so it was possible to submit partially completed questionnaires. Participants were asked to rate the medical adequacy of the 2 recommendations made by the respective LLMs on a 6-point Likert scale (1=“very poor” and 6=“excellent”). Moreover, MDT members were further asked whether the LLM’s response could improve the board’s decision (yes/no) and whether the respective LLM response was potentially hazardous for the patient (yes/no). “Hazardous to patients” was defined as the presence of information that could directly or indirectly cause harm to the patient if followed. This includes advice that contradicts established clinical guidelines, promotes unsafe practices, misrepresents risks or benefits, or could lead to delayed diagnosis, inappropriate treatment, or adverse outcomes. Additionally, MDT members were provided with the chance to give written feedback on the respective LLM recommendations. Beyond the rating of the MDT members, the therapy regimens of the MDT and the 2 LLMs were compared in a table for concordance.

### Outcomes

The primary outcome was the proportion of cases in which the MDT recommendation changed after review of the LLM outputs. Secondary outcomes were (1) concordance with MDT treatment intent (curative vs palliative), (2) overlap with the MDT first-line regimen, (3) MDT member ratings of medical adequacy, perceived potential benefit, and potential hazard, and (4) qualitative themes from free-text comments.

### LLM Execution

Information from the regular MDT registration, including diagnosis, tumor-node-metastasis stage, date of initial diagnosis, imaging reports, pathology reports, previous treatment, secondary diagnoses, comments, and the query to the MDT, was transferred to a Word document (Microsoft Word) and presented to the nongrounded, locally run LLMs using the prompt shown in Figure 2 (for the full prompt, see Multimedia Appendix 1). LLM results were retrieved based on a single-shot strategy. The LLMs were run locally on a standard Hewlett Packard (Palo Alto, CA, USA) notebook (Intel Core i7-1255U, 4.7 GHz; DDR4, 16 GB [2×8 GB], Windows 10 Pro; LM Studio version 0.3.15/23). The selection of LLMs was guided by their open-weight availability and model size. Consequently, only models with below 13 GB were included. The default settings of LM Studio were not modified. All model configurations—including parameter sizes or variants, quantization settings, prompt templates and system prompts, as well as temperature, top-p, maximum token limits, and random seeds—were kept unchanged. The specific models evaluated in this study are as follows: lmstudio-community/gemma-3-12b-it-gguf/gemma-3-12b-it-q3_k_l.gguf (GPU offload 33/42; CPU thread pool size 4; evaluation batch size 512; temperature 0.1; top-*p* sampling 0.95; context length 4096; random seed) and lmstudio-community/gpt-oss-20b-gguf/gpt-oss-20b-mxfp4.gguf (GPU offload 11/40; CPU thread pool size 4; evaluation batch size 512; temperature 0.8; top-*p* sampling 0.95; context length 4096; random seed; all published by lmstudio-community).

### Statistical Analysis

All responses from the raters were transferred to an Excel spreadsheet (Microsoft Excel) and sorted according to the entity being evaluated. The statistical analysis was performed using GraphPad Prism software (version 10.6.1 for macOS; GraphPad Software). The data did not meet normality assumptions, as confirmed by the D’Agostino and Pearson test (ns: *P*>.05, *P<.05, **P<.005, ***P<.0005). Differences regarding the ratings for medical adequacy (6-point Likert scale) between LLMs were analyzed by the Wilcoxon matched-pairs signed-rank test. The word count among different LLM answers was analyzed by the Friedman test and Dunn multiple comparison post hoc test. Binary-rated categories were analyzed for significant differences using exact McNemar test in SAS software (version 9.4 for Windows; SAS Institute Inc). The 95% CIs of proportions were calculated by the Clopper-Pearson method using the Ausvet 2026 Epitools CI Calculator [14].

MDT members completed anonymous questionnaires independently, either digitally or on paper. As participation varied between cases and questionnaires did not contain mandatory fields, the number of ratings differed across patients and models, resulting in an incomplete and unbalanced dataset without persistent rater identifiers. Therefore, formal interrater reliability statistics requiring fixed rater assignments across items (eg, Fleiss κ or the intraclass correlation coefficient) were considered methodologically inappropriate. Instead, rating consistency was assessed descriptively for each patient case and LLM separately using the number of ratings, median, IQR, range, and the proportion of the most frequent rating. Analysis and data processing for this part of the analysis were performed using Python in Google Colab to ease the implementation.

### Qualitative Rating by MDT Members and Post Hoc Analysis of LLMs Recommendations

The comments of the MDT members were collected in a Word document and summarized. Additionally, the authors’ observations during the tabular comparison of concordance in therapy regimens were also summarized and kept on record.

### Data Privacy

All data were processed on locally run entities, preserving the data privacy of patients. The data underlying this paper cannot be shared publicly to protect the privacy of the individuals who participated in the study.

## Results

### Relevant Amendment of MDT Recommendation

In 1 case, the LLM (gemma-3-12b) pointed out a secondary finding that had been noticed during staging (computed tomography scan) but had not yet been checked. In this case, the MDT’s recommendation was substantially amended with a corresponding note regarding a follow-up examination.

### Concordance in Therapy Regimen

Recommended therapy regimens generated by the MDT and the different LLMs are illustrated in Tables 1 and 2. While the MDT recommended a curative treatment regimen for 88% (15/17, 95% CI 64%‐99%) of patients, gemma-3-12b recommended this for 82% (14/17, 95% CI 57%‐96%), and gpt-oss-20b for 59% (10/17, 95% CI 33%‐82%). This corresponds to 94% (16/17, 95% CI 71%‐100%) agreement between gemma-3-12b and the MDT and a 59% (10/17, 95% CI 33%‐82%) agreement between gpt-oss-20b and the MDT. For 35% (6/17, 95% CI 14%‐62%) of patients, gemma-3-12b stated the same first-line therapy regimen as the MDT as first-line, with gpt-oss-20b achieving the same for 41% (7/17, 95% CI 18%‐67%) of patients. In 59% (10/17, 95% CI 33%‐82%) of patients, gemma-3-12b stated the MDT’s first-line therapy regimen, albeit with a different priority, while in gpt-oss-20b, it was 41% (7/17, 95% CI 18%‐67%) of patients. For a further 24% (4/17, 95% CI 7%‐50%) of patients, gemma-3-12b and for 47% (8/17, 95% CI 23%‐72%) of patients, gpt-oss-20b stated parts of the first-line therapy regimen recommended by the MDT.

**Table 1.** Comparison of curative and palliative therapy regimens (N=17).

	MDT^a^, n (%; 95% CI)	gemma-3-12b, n (%; 95% CI)		gpt-oss-20b, n (%; 95% CI)	
		Recommendation	Concordance with MDT	Recommendation	Concordance with MDT
Therapy regimen			16 (94; 71‐100)		10 (59; 33‐82)
Curative therapy regimen	15 (88; 64‐99)	14 (82; 57‐96)		10 (59; 33‐82)	
Palliative therapy regimen	2 (12; 1‐36)	3 (18; 4‐43)		0 (0; 0‐20)	

a MDT: multidisciplinary tumor board. *For 7 cases, gpt-oss-20b provided no clear treatment recommendation and instead suggested additional examinations.

**Table 2.** First-line therapy regimens recommended by the large language models compared with the multidisciplinary tumor board (N=17).

Recommendation	gemma-3-12b, n (%; 95% CI)	gpt-oss-20b, n (%; 95% CI)
Stated all first-line therapy regimens of the MDT^a^	10 (59; 33‐82)	7 (41; 18‐67)
Stated some first-line therapy regimens of the MDT as first-line	4 (24; 7‐50)	8 (47; 23‐72)
Stated all first-line therapy regimens as first-line, consistent with the MDT	6 (35; 14‐62)	7 (41; 18‐67)

a MDT: multidisciplinary tumor board.

### Quantitative Rating by MDT Members

**Figure 3.**
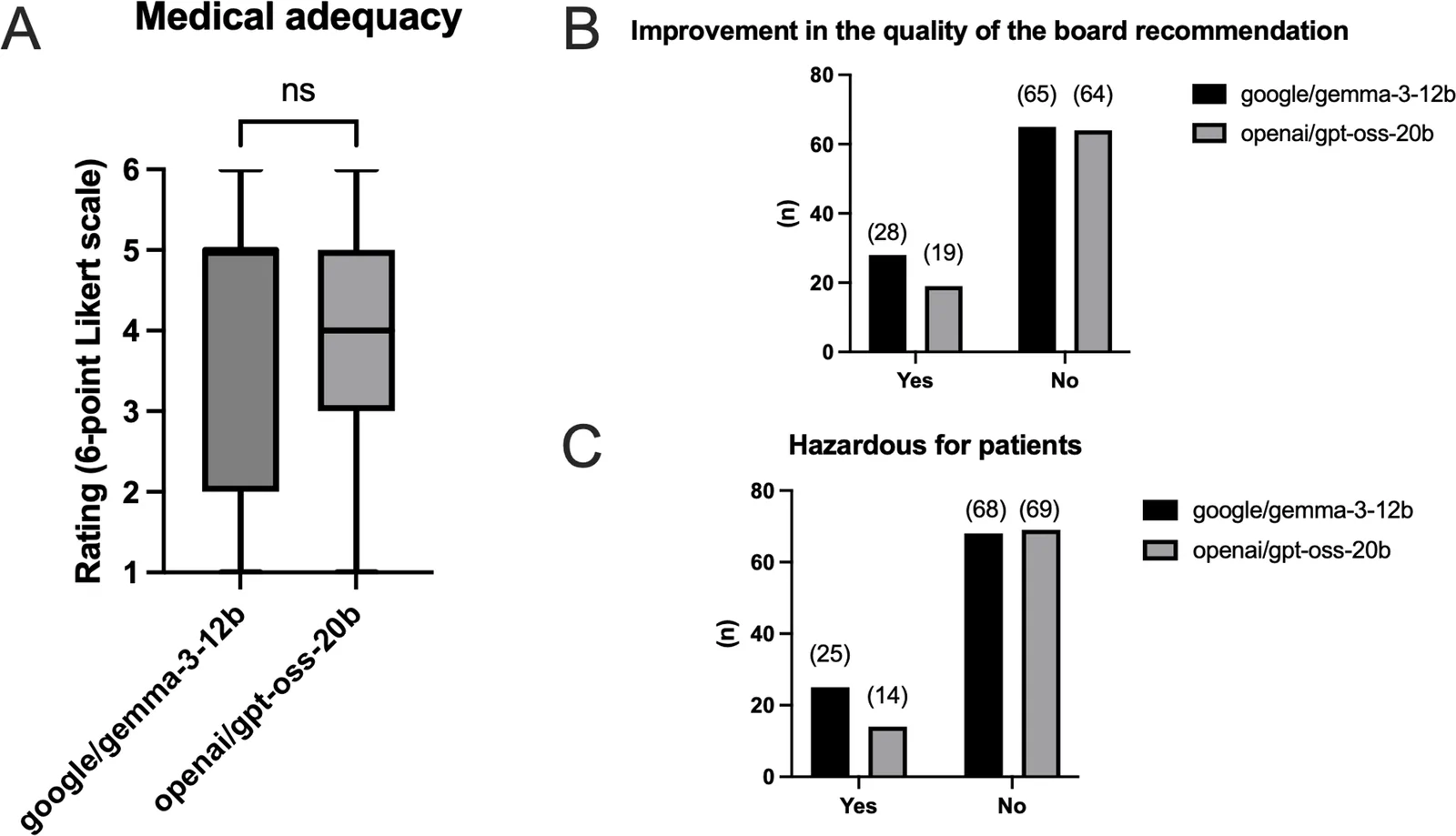
Quantitative rating by multidisciplinary tumor board members. Medical adequacy rated on a 6-point Likert scale (A), shown as a box plot. Improvement of the board recommendation (B) and presence of information potentially hazardous for patients (C), shown as binary outcomes in absolute numbers (n). Differences between the LLMs were tested using the Wilcoxon test for medical adequacy (ns; *P=0.8094*) and the exact McNemar test for ‘Improvement in the quality of the board recommendation’ (B) and ‘Potentially hazardous information’ (C). The difference was not statistically significant for improvement of the board recommendation (P=0.0707) but was statistically significant for potentially hazardous information (P=0.0330).

Medical adequacy, as rated by the MDT members, revealed a median rating of 5 (IQR 2‐5) for gemma-3-12b and a median of 4 (IQR 3‐5) for gpt-oss-20b (Figure 3A). The MDT members stated that the information provided by gemma-3-12b had the potential to improve the MDT decision in 30% (28/93, 95% CI 21%-40%) of ratings. For gpt-oss-20b, this was stated in 23% (19/83, 95% CI 14%‐33%) of ratings. Regarding potential hazards for patients, MDT members stated potentially hazardous information in 27% (25/93, 95% CI 18%-37%) of ratings for gemma-3-12b and 17% (14/83, 95% CI 9%-26%) of ratings for gpt-oss-20b. No significant difference (*P=0.8094*) between the LLMs was found for medical adequacy according to the Wilcoxon test (Figure 3A). Regarding improvement of the board recommendation (Figure 3B), no significant difference was observed between the LLMs (exact McNemar test, P=0.0707). In contrast, a statistically significant difference was observed for potentially hazardous information (Figure 3C), with hazardous information reported in 27% (25/93) of ratings for gemma-3-12b compared with 17% (14/83) for gpt-oss-20b (exact McNemar test, P=0.0330)t.

### Qualitative Rating by MDT Members and Post Hoc Analysis of LLMs Recommendations

The LLM integration was beneficial in verifying the completeness of MDT submissions. However, some recommendations of the LLMs also indicated potential hazards for patients. Therefore, the free-text comments of MDT members indicating potential hazards were systematically categorized into factual errors, misinterpretation of the case, reasoning errors, and semantic errors (Table S1 in Multimedia Appendix 1). For both LLMs, the MDT members stated factual errors for 4 patients each. Misinterpretation of the case was found in 3 cases for gemma-3-12b and 1 case for gpt-oss-20b. Reasoning errors were highlighted in 2 cases for gemma-3-12b and 1 case for gpt-oss-20b. Moreover, the MDT emphasized semantic errors in 1 case for gpt-oss-20b and none for gemma-3-12b.

Factual errors often concerned decisions regarding adjuvant treatment. In cases where the LLMs’ recommendations differed from the MDT’s decisions, both directions were present: either no recommendation for adjuvant therapy despite the presence of risk factors (undertreatment) or a recommendation for adjuvant therapy in the absence of risk factors (overtreatment).

Although general information, such as the reference to resection margins, was presented to the LLMs, in some cases the LLMs lost track of the full clinical picture. For example, sample collection by panendoscopy was classified as an R1 resection, or a tonsillectomy performed as part of a diagnostic panendoscopy was classified as a curatively intended resection of the tumor.

However, MDT members frequently felt that the reasoning behind the LLMs’ treatment recommendations was incorrect. In one example, the indication for radiotherapy was based solely on the resection status, even though the indication was already present due to the tumor (T) stage. Furthermore, the LLMs sometimes did not include important information, such as prior exposure to radiotherapy, in the decision-making process, even though this information was evident from the documents provided.

In some cases, new words were created by the LLMs, such as the description of a “pulmocarcinoma.” There was also confusion regarding the distinction between neoadjuvant, primary, and adjuvant radio(chemo)therapy. In some cases, the therapy recommended by the LLM could be deduced from the context, but in others it remained unclear what the LLM was exactly referring to. The LLMs seemed to lose track, particularly in complex cases where additional tumors were present, such as a parallel secondary carcinoma of the lung.

It was also noticeable that gpt-oss-20b showed a marked preference for PET-CTs (positron-emission-tomography-computed tomography). While a PET-CT was performed before the MDT in only 1 case, and a PET-CT was recommended by the MDT as the next diagnostic step for another patient, gpt-oss-20b mentioned PET-CT for 8 patients in its recommendation. In a further 5 patients, gpt-oss-20b insisted on performing a PET-CT scan before providing further recommendations. In only 4 patients did gpt-oss-20b not mention a PET-CT scan at all. In contrast, gemma-3-12b only considered a PET-CT scan in 3 patients. In one of these patients, the MDT recommended the same, and another patient received a PET-CT ahead of the MDT. Some MDT members criticized the fact that the LLM (gpt-oss-20b) frequently required a PET-CT scan and did not discuss any further treatment steps. It was pointed out that the LLMs should provide treatment plans for different investigation outcome scenarios in order to reduce further delay in treatment.

### Number of Words

**Figure 4.**
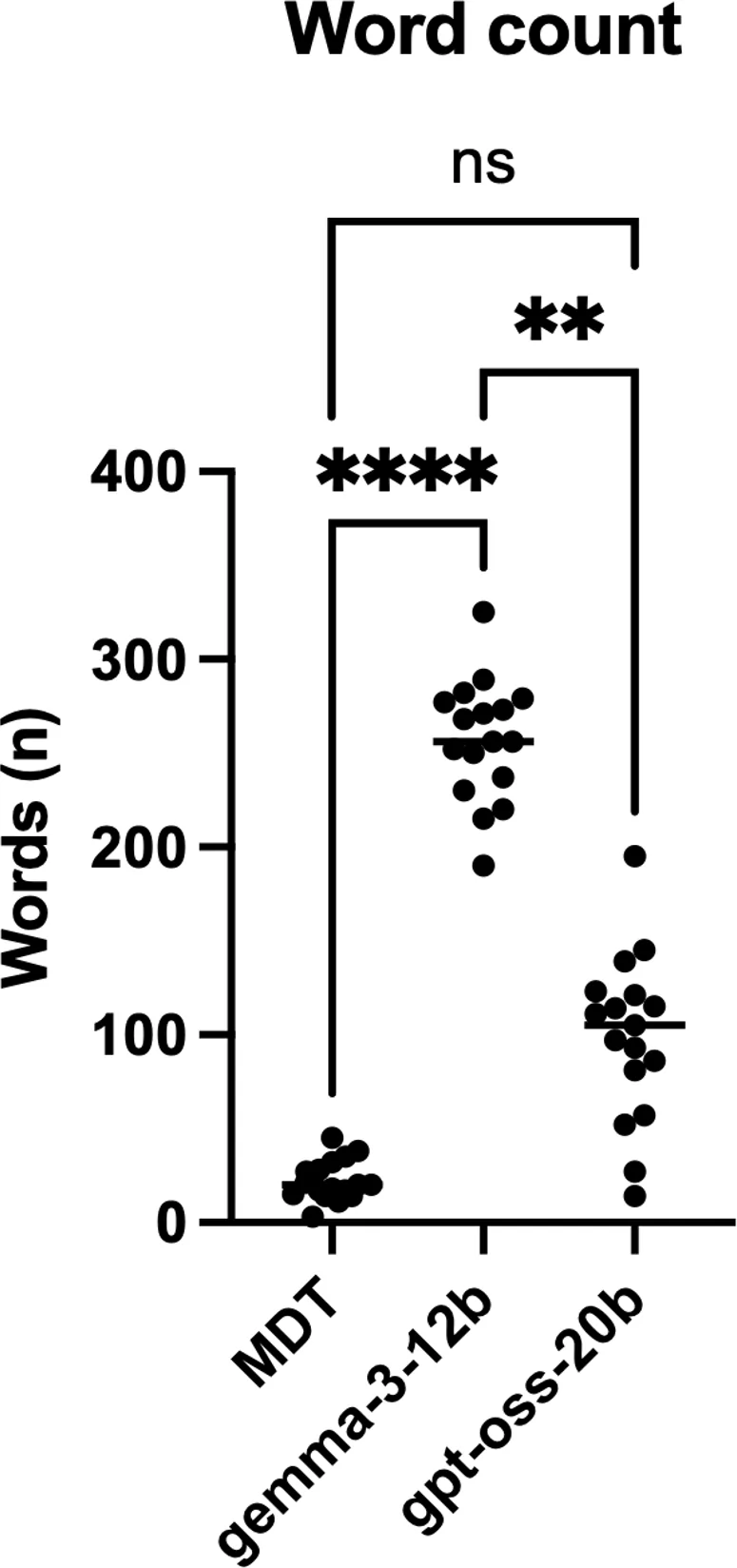
Word count of the recommendations stated by the MDT, gemma-3-12b, and gpt-oss-20b. A Friedman test showed significant differences in word count between the entities (*P*<.001). Post hoc analysis was performed using the Dunn multiple comparison test (ns: *P*>.05; **P<.005; ****P<.001). MDT: multidisciplinary tumor board.

The word count of the recommendations provided by the MDT and the LLMs is visualized in Figure 4. While gemma-3-12b showed the highest word count, with a median of 256 (IQR 233.5‐278) words, gpt-oss-20b used a median of 105 (IQR 69‐122) words, and the MDT a median of 20 (IQR 14.5‐30) words. Intergroup comparison in the Friedman test showed significant differences (*P*<.001) among groups. The post hoc analysis using Dunn multiple comparison test revealed significant differences between the MDT and the gemma-3-12b (*P*<.001), as well as between gemma-3-12b and gpt-oss-20b (*P*<.005). No significant differences were found between the MDT and gpt-oss-20b (*P*>.05).

### IDescriptive Analysis of Interrater Agreement

The number of MDT members ranged from 8 to 12 (median 11, IQR 11‐12), and the LLMs received a median number of 6 (IQR 4‐6) ratings per case. The within-case descriptive agreement analysis demonstrated heterogeneous rating dispersion across cases and models. Several cases showed narrow IQRs and high proportions of identical ratings, indicating substantial agreement among MDT participants. However, other cases demonstrated broader rating distributions and wider ranges, reflecting greater variability in expert assessment. Overall, the analysis suggested that agreement was case-dependent rather than uniformly high or low across all evaluations. For detailed information, see Table S2 of Multimedia Appendix 1 (within-case variability of expert Likert ratings).

### Implications for Further Studies

A power analysis was carried out using the exact McNemar test to determine the sample sizes required for future studies. Based on 80% power, a total of 240 patients (5% vs 13% discordant pairs) would be required to detect an improvement in the board recommendation outcome. Regarding the rating of potential hazard to patients, a total of 150 patients (7% vs 19% discordant pairs) would be required to achieve 80% power at a 5% significance level.

## Discussion

### Contextualization

Although the application of LLMs for MDT augmentation has been extensively evaluated, previous monocenter simulation (in silico) studies in head and neck cancer focused primarily on web-based LLMs [1-3,8]. Only 2 studies tested the performance of locally run LLMs in head and neck cancer MDTs. However, these studies used constructed cases rather than real-world data [7] or had a retrospective design [8]. Accordingly, this is the first prospective study of its kind to use real-world MDT augmentation by guideline-instructed, nongrounded, locally operated LLMs in the field of head and neck oncology. Much of the current literature remains in silico. LLMs are evaluated on simulated vignettes or retrospectively curated cases, often with more complete information than is available at the time of a live MDT. By contrast, real-world prospective testing captures practical constraints such as incomplete referrals, time pressure, and the need for short, actionable outputs, and therefore provides a more stringent assessment of clinical utility and risk.

### Performance of Locally Operated LLMs

Within this study, gemma-3-12b showed 94% (16/17, 95% CI 71%‐100%) and gpt-oss-20b showed 59% (10/17, 95% CI 33%‐82%) concordance with the MDT regarding the curative or palliative therapy regimen (Table 1). For 7 cases of gpt-oss-20b no clear recommendation was provided and additional examination was suggested. In similar preliminary work with simulated cases in the head and neck region, 92% concordance was reported for Llama 3 and 84% concordance for ChatGPT-4o [7]. Accordingly, Gemma-3-12b achieves slightly higher concordance values than those reported in the literature, while gpt-oss-20b achieves lower values. Albeit with a different priority, gemma-3-12b stated the same first-line therapy regimen as the MDT in 59% (10/17, 95% CI 33%‐82%) of cases, while gpt-oss-20b reached 41% (7/17, 95% CI 18%‐67%) concordance with the MDT. Our previous study evaluating constructed cases showed 64% concordance for ChatGPT-4o and 60% for Llama 3 [7]. In 35% (6/17, 95% CI 14%‐62%) of cases, gemma-3-12b stated the same first-line therapy regimen as the MDT as first-line therapy, whereas gpt-oss-20b achieved 41% (7/17, 95% CI 18%‐67%) concordance (Table 1). Here, both LLMs tested in this study performed worse than the published performance of ChatGPT-4o (52%) and Llama 3 (48%) [7].

Regarding medical adequacy, gemma-3-12b reached a median rating of 5 (IQR 2‐5) and gpt-oss-20b a median of 4 (IQR 3‐5) in this study. Here, no significant difference (*P*>.05) between the LLMs was found (Figure 3A). While gemma-3-12b outperformed published values of ChatGPT-4o (median 4.7, IQR 4‐6) and Llama 3 (median 4.3, IQR 3‐5), gpt-oss-20b underperformed compared with the published benchmark [7].

MDT members found the LLMs to have the potential to improve MDT decisions in 30% (28/93, 95% CI 21%‐40%) for gemma-3-12b and 23% (19/83, 95% CI 14%‐33%) for gpt-oss-20b (Figure 3B). Here, in the previous study, the MDT members stated the same in 17% of ratings [7]. With regard to potential hazards for patients, MDT members reported potentially hazardous information in 27% (25/93, 95% CI 18%‐37%) of ratings for gemma-3-12b and 17% (14/83, 95% CI 9%‐26%) of ratings for gpt-oss-20b. This difference was statistically significant (exact McNemar test, P=0.0330) highlighting that differences between locally run LLMs may not only concern the quality or completeness of their recommendations but also their safety profile.

Within the prompt, both LLMs were instructed to limit their response to 100 words (Figure 2). While gpt-oss-20b adhered to the word limit fairly well (median 105, IQR 69‐122), gemma-3-12b deviated from it (median 256, IQR 233.5‐278) and showed a significant deviation (*P*<.001) from the MDT’s response length (median 20, IQR 14.5‐30; Figure 4).

### Qualitative Performance of the LLMs

The qualitative analysis of the LLM recommendations highlights the current inability of the tested LLMs to generate comprehensive therapy recommendations similar to those of an MDT. The MDT members felt the LLMs offered incorrect justifications for therapy recommendations, confusion regarding nomenclature (eg, in distinguishing between neoadjuvant, primary, and adjuvant radio(chemo)therapy), and the misunderstanding that a panendoscopy with sample collection does not yet include surgical resection. Furthermore, the LLMs created surprising neologisms such as “pulmocarcinoma.” These errors can be considered as a form of LLM “hallucination” [15]. Although gpt-oss-20b has a reasoning mechanism, these errors occurred in both LLMs. Such neologisms arise because mid-sized local LLMs recombine meaningful medical morphemes when they lack strong domain-specific lexical constraints. This reflects limited specialization and alignment rather than random error.

Another phenomenon was the tendency of the LLMs to suggest further examinations. For instance, gpt-oss-20b recommended PET-CT for multiple patients. This phenomenon was further underscored by the fact that gpt-oss-20b never recommended palliative treatment and instead suggested to broaden the diagnostic scope. The tendency of LLMs to demand more diagnostics than doctors has been described in previous work [1] and might be justified by their focus on safety. Nonetheless, additional examinations are accompanied by risks such as radiation exposure or delayed time to treatment and incur relevant resources. This behavior stems from LLMs’ being trained to maximize uncertainty reduction and patient safety, which biases them toward recommending additional diagnostic tests. Unlike clinicians, the models do not directly incorporate real-world constraints such as workflow pressure, resource availability, and patient-specific feasibility unless these are explicitly represented in the input.

### Analysis of Interrater Agreement

The observed variability in expert ratings likely reflects the inherent complexity and interpretative nature of multidisciplinary oncological decision-making. While some LLM-generated recommendations were evaluated consistently by MDT participants, other cases elicited broader disagreement, potentially reflecting differing clinical perspectives among specialties. Due to the anonymous and incomplete survey structure, formal inter-rater reliability statistics could not be robustly applied. Nevertheless, the case-based descriptive agreement analysis provides transparent insight into the consistency and dispersion of expert evaluations within the study cohort.

### Implications of the Study

To our knowledge, there are no studies investigating the prospective use of locally operated LLMs in head and neck cancer. The present study evaluates the feasibility of integrating locally operated LLMs in today’s MDT process. In clinical practice, MDT submissions are checked for completeness and accuracy by a doctor before every MDT. Within our study, this preprocessing took around 15 minutes per case, including processing by both tested LLMs. Accordingly, the extra time required is only a few minutes per case. This also highlights a positive aspect of LLM integration: LLMs can assist during this process, for example, by helping to identify inconsistencies in the submissions or pointing out missing data or diagnostic information. The presentation and discussion of the LLMs’ recommendations within the MDT took only a few minutes per case. This workflow was appreciated by the participants and received with interest. However, in this study, we have limited the discussion to the outputs of two LLM cases in order to avoid an overload of the MDT meeting.

Presenting the LLM outputs after the MDT decision may introduce some degree of bias. In this study, this specific workflow was required due to ethical considerations, as the MDT’s decision should not be primarily influenced by the LLMs. One could argue that, given the sequence of events, doctors might find it more difficult to deviate from the decision that has been made. However, within our study, the recommendation was adjusted by the LLM in 1 case, as mentioned above. On the contrary, the applied order reduces the risk of automation bias because the MDT finds consensus before knowing the LLMs’ recommendation. Automation bias occurs when doctors rely too heavily on AI decisions [16].

Evaluating the models using a single-shot strategy, their respective default LM Studio inference configurations and quantization schemes, and default settings is consistent with a real-world setup. However, differences in temperature and quantization may have influenced output determinism and reasoning performance. Therefore, results should be interpreted within the context of practical deployment settings rather than fully standardized inference conditions.

The statistical analyses did not explicitly account for clustering, as multiple ratings were obtained per patient case and LLM response. Because ratings were collected anonymously without persistent reviewer identifiers, retrospective modeling of rater-level clustering using mixed-effects models or generalized estimating equations was not feasible. This may have affected the precision of *P* values and confidence intervals. Therefore, the reported inferential statistics should be interpreted with appropriate caution.

We deliberately evaluated 2 small open-weight models (12B and 20B parameters) that can run on standard local hardware to maximize feasibility, decentralization, and data protection by keeping all processing behind the institutional firewall. This design contrasts with cloud-based, full-size proprietary LLMs (“cloud full LLMs”), which may offer higher performance but typically require the transfer of patient data to an external provider.

An intermediate deployment option is large open-weight models self-hosted on institutional GPU servers. Such models may narrow the performance gap with cloud-based systems while maintaining on-premises data control, but they increase infrastructure requirements and may be less accessible for smaller centers. Checking for completeness also proved to be a major advantage of the LLMs, showcasing a promising use case for LLM implementation. Here, LLMs supporting the MDT registration process can identify missing examinations prior to the MDT, avoiding unnecessary delays in therapy [17].

Although the treatment regimens between gemma-3-12b and the MDT matched in 35% (6/17, 95% CI 14%‐62%) of patients and between gpt-oss-20b and MDT in 41% (7/17, 95% CI 18%‐67%) of patients, the recommendations of the LLMs were not able to replace the MDT recommendation in these patients. The reasons for this are various minor errors in the justification for the therapy or an incorrect summary of the case (panendoscopy with sample collection being considered a surgical resection).

Nevertheless, MDT members found the recommendations of the LLMs helpful in 30% (28/93, 95% CI 21%‐40%) for gemma-3-12b and 23% (19/83, 95% CI 14%‐33%) for gpt-oss-20b. This suggests that carefully supervised real-world implementation of LLMs may provide useful adjunctive support in MDT workflows, rather than augmenting or replacing the MDT’s opinion.

Larger LLMs running on large servers are likely to provide improved results, and further development and improvement of the models may additionally lead to more suitable recommendations. Other enhancement opportunities are fine-tuning [8] and grounding the LLMs based on relevant guidelines and current studies [8,18]. Importantly, our approach was guideline-instructed rather than guideline-grounded. The LLMs were prompted to follow a guideline hierarchy and to cite sources, but were not provided with the guideline documents themselves. Guideline-grounded systems that retrieve and supply the relevant guideline passages at inference time could improve factuality, enable auditable citations, and reduce hallucinated or outdated recommendations. The frequency of potentially hazardous content observed in this study supports prioritizing such grounding in future implementations. Both approaches should be further evaluated in future studies in head and neck. Ideally, an “in the loop” process is being established in which LLMs are applied, supervised by doctors, and continuously optimized in appropriate centers.

Further improvements may be achieved through domain-specific vocabulary constraints, retrieval-augmented generation to integrate up-to-date guidelines and clinical evidence at inference time [19], and recursive language models enabling iterative refinement of clinical reasoning [20].

Rating medical adequacy, potential improvement, and hazard for patients can highly be affected inter alia by the personal views, experience, and last but not least, the subspecialty of the raters (MDT members). However, this is a general problem of evaluating LLMs. In order to address this issue, we analyzed the LLMs’ recommendations for concordance with the MDT recommendation. The MDT found consensus before knowing the LLMs’ recommendation to avoid influence. The MDT itself is an organ of internal validation as interdisciplinary perspectives on treatment are bundled in a meeting to combine the expertise and validate the treatment approach. Furthermore, our university hospital is a German Cancer Society–validated tumor center and thus subject to external validation on a regular basis. The comparison for concordance with the MDT was implemented in order to provide a more objective assessment. However, even the definition of concordance is prone to bias of subjectivity. Our study was specifically designed to test a use case that could be implemented straightforwardly in a clinical setting. The more complex the issue and the case, the more complex the assessment. When it comes to cancer patients’ treatment plans, there might be more than a single correct recommendation, despite the existence of oncological guidelines. Treatment strategies depend on a great variety of different nuances, due to risk factors, comorbidities, and the patient’s preferences, as well as the center’s expertise or regional specifications. Other use cases for LLMs, such as determining clearly defined tumor stages based on imaging findings, are significantly easier to evaluate [21]. As the perception of LLMs is also highly relevant, we have deliberately opted for a subjective criterion assessed by the MDT participants (experts) and a comparison with a more objective criterion: the MDT’s recommendation (the current gold standard). At present, we consider this approach to be sensible also for other specialist fields beyond otorhinolaryngology-head and neck surgery. This may change in the future. The evaluation of LLMs themselves also offers plenty of scope for future research.

### Ethical Considerations

An application of AI in clinical practice is subject to ethical discussions. Despite the obvious shortcomings of the LLMs in this study, in one specific case, the LLM made a relevant difference by pointing out that a further examination was pending. This examination might have been overlooked without the LLM’s recommendation. This instance raises a central question: Is it ethically justifiable not to use AI in the clinic, even if—as in this study—it only makes a relevant difference for one patient? Of course, it is not a question of replacing the doctor; the local LLMs in this study have proven that they are not capable of replacing human medical recommendations. Beyond measurable criteria for decision-making, being a doctor includes interpersonal relationships and the “soft parameters,” which cannot be replaced by AI. However, perhaps AI should be implemented as an additional tool in general, even if, at the current stage, it is only to check for completeness, which may not always be checked with absolute accuracy in the hurry of everyday clinical practice.

### Conclusion

This first prospective study demonstrates the feasibility of an auxiliary implementation of LLMs in today’s MDT’s workflow. The low-barrier setup using small, locally run, open-weight LLMs suitable for standard computers can have a positive influence on MDT decisions in individual cases. However, rather than substantial improvement of MDT decisions, the benefits of the LLMs´ recommendations were mainly limited to the indication of follow-up examinations that are at risk of being lost within the flood of information. Nonetheless, implementation of LLMs as an augmentation of MDT decision-making is still promising. First, even occasional, and seemingly modest improvements of MDT recommendations, like the reminder regarding a pending follow-up examination, could significantly influence the fate of a specific patient. Second, the performance of (locally run) LLMs will further improve in the foreseeable future. More advanced models will very likely offer further improvements in more aspects of recommendation. Beyond checking the completeness of the provided data and planned examinations, LLMs may support the development of individualized treatment concepts based on recent studies that have not yet been incorporated into clinical guidelines. Furthermore, suitable LLMs may streamline patients’ inclusion in appropriate prospective experimental treatment studies [22-24]. Future studies should focus on the use of locally operated LLMs with the potential data protection advantages of local deployment and greater likelihood of being implemented within existing clinical IT infrastructure. Although multiple retrospective studies evaluating the use of LLMs in MDTs have been published, prospective studies, particularly those evaluating locally run LLMs, remain limited. The present study therefore contributes to the emerging evidence on prospective implementation of locally run LLMs and aims to pave the way for pragmatic implementation in real clinical practice.

## Supplementary material

10.2196/95081 Multimedia Appendix 1 Prompt (translated to English with DeepL.com).

## Abbreviations

GPU: graphics processing unit
LLM: large language model
MDT: multidisciplinary tumor board
