# Supplementary material for: Augmenting Head and Neck Multidisciplinary Tumor Board Recommendations With Locally Run Large Language Models: Prospective Evaluation of Real-World Implementation
Source: JMIR AI. 2026 Aug 20;5:e95081. doi: 10.2196/95081 (PMC13492482; doi:10.2196/95081)
Supplement: Supplementary file 1 [file ai-v5-e95081-s001.docx]

**Prompt (Translated to English with DeepL.com)**

[Role & Context]

Virtual tumour board for head and neck tumours (ENT, maxillofacial surgery, oncology, radiology, radiotherapy, pathology).

[Guideline hierarchy]

1. German S3 guideline

2. If not available: ESMO

3. NCCN only as a supplement, clearly marked as ‘(NCCN)’

Always mark optional recommendations with ‘optional’.

[Task]

1. Check: Are all guideline-mandated diagnostic steps present?

- No → only list missing diagnostics, end response.

2. If diagnostics are complete:

- Treatment status “completed” → follow-up care recommendation including timetable

- Treatment status “open” → treatment recommendation with justification

3. If alternatives exist: specify equivalence and conditions of use.

[Output format] (max. 100 words)

1. Summary (2–3 sentences)

2. Missing diagnostics (“none” if complete)

3. Treatment status

4. Primary treatment recommendation (only if status is open)

5. Alternative options (including equivalence)

6. Follow-up care (only if status is completed)

7. Open questions

8. Sources

9. Guideline compliance (guideline used + chapter/page; NCCN only supplementary)

[Patient data]

Diagnosis:

Initial diagnosis: (date)

cTNM at initial diagnosis:

ECOG:

Presentation for: (Initial diagnosis/Locoregional recurrence/Metastasis/Course of treatment/Other)

Histology:

Imaging:

Treatment and course:

Relevant secondary diagnosis:

Comments (if applicable):

Clinical question:

**Table 1: Categorization of hazards stated by MDT members**

| **Categorization of hazards stated by MDT members** | | |
| --- | --- | --- |
|  | **gemma-3-12b** | **gpt-oss-20b** |
| **Factual errors** | 4 | 4 |
| **Misinterpretation of the case** | 3 | 1 |
| **Reasoning errors** | 2 | 1 |
| **Semantic errors** | 0 | 1 |

**Table 2: Within-case variability of expert Likert ratings**

| **Fall** | **LLM** | **n_ratings** | **median** | **IQR** | **Range** | **agreement_percent** |
| --- | --- | --- | --- | --- | --- | --- |
| 1 | Gemma | 8 | 5.000 | 5.000–5.250 | 4–6 | 62.500 |
| 1 | GPT | 6 | 5.000 | 4.250–5.000 | 2–6 | 50.000 |
| 2 | Gemma | 4 | 1.000 | 1.000–1.000 | 1–1 | 100.000 |
| 2 | GPT | 3 | 6.000 | 5.500–6.000 | 5–6 | 66.667 |
| 3 | Gemma | 4 | 5.500 | 5.000–6.000 | 5–6 | 50.000 |
| 3 | GPT | 4 | 4.000 | 3.750–4.500 | 3–6 | 50.000 |
| 4 | Gemma | 5 | 5.000 | 5.000–5.000 | 5–6 | 80.000 |
| 4 | GPT | 5 | 4.000 | 3.000–5.000 | 2–5 | 40.000 |
| 5 | Gemma | 4 | 4.000 | 2.750–5.250 | 2–6 | 25.000 |
| 5 | GPT | 3 | 3.000 | 2.500–4.000 | 2–5 | 33.333 |
| 6 | Gemma | 6 | 2.000 | 2.000–3.500 | 2–4 | 66.667 |
| 6 | GPT | 5 | 3.000 | 3.000–4.000 | 2–4 | 40.000 |
| 7 | Gemma | 6 | 5.000 | 2.750–5.000 | 2–6 | 50.000 |
| 7 | GPT | 5 | 4.000 | 3.000–5.000 | 2–5 | 40.000 |
| 8 | Gemma | 6 | 4.500 | 3.250–5.750 | 3–6 | 33.333 |
| 8 | GPT | 6 | 3.000 | 2.250–3.750 | 2–5 | 33.333 |
| 9 | Gemma | 6 | 2.000 | 1.250–2.750 | 1–6 | 33.333 |
| 9 | GPT | 6 | 6.000 | 5.250–6.000 | 5–6 | 66.667 |
| 10 | Gemma | 6 | 5.000 | 5.000–5.750 | 4–6 | 50.000 |
| 10 | GPT | 5 | 2.000 | 2.000–4.000 | 1–6 | 40.000 |
| 11 | Gemma | 5 | 5.000 | 5.000–6.000 | 4–6 | 40.000 |
| 11 | GPT | 5 | 5.000 | 4.000–6.000 | 4–6 | 40.000 |
| 12 | Gemma | 4 | 5.000 | 4.500–5.000 | 3–5 | 75.000 |
| 12 | GPT | 4 | 3.000 | 3.000–3.500 | 3–5 | 75.000 |
| 13 | Gemma | 6 | 4.000 | 3.250–4.750 | 2–5 | 33.333 |
| 13 | GPT | 6 | 4.500 | 4.000–5.000 | 2–6 | 33.333 |
| 14 | Gemma | 4 | 4.000 | 2.000–6.000 | 2–6 | 50.000 |
| 14 | GPT | 4 | 3.000 | 2.000–4.500 | 2–6 | 50.000 |
| 15 | Gemma | 4 | 3.000 | 1.750–4.250 | 1–5 | 25.000 |
| 15 | GPT | 4 | 3.000 | 1.750–4.250 | 1–5 | 25.000 |
| 16 | Gemma | 6 | 3.000 | 2.000–5.500 | 2–6 | 50.000 |
| 16 | GPT | 6 | 5.000 | 4.250–5.000 | 3–6 | 50.000 |
| 17 | Gemma | 5 | 4.000 | 4.000–4.000 | 4–5 | 80.000 |
| 17 | GPT | 5 | 3.000 | 3.000–3.000 | 2–6 | 60.000 |
